# Esophageal Actinomycosis Presenting as an Obstructive Esophageal Mass

**DOI:** 10.14309/crj.0000000000000140

**Published:** 2019-07-23

**Authors:** Ali Alshati, Leslie Appleton, Jacob T. Maddux, Mays Almohammedawi, Benjamin Dangerfield

**Affiliations:** 1 Department of Internal Medicine, Creighton University School of Medicine, Maricopa Medical Center, Phoenix, AZ; 2 Department of Internal Medicine, College of Medicine, University of Arizona, Phoenix, AZ; 3 Department of Internal Medicine, Mayo Clinic School of Medicine, Phoenix, AZ; 4 Department of Medicine, Baghdad University, College of Medicine, Baghdad, Iraq

## Abstract

Esophageal actinomycosis is a rare type of esophageal infection, with only approximately 24 cases previously reported in the United States. Most of these cases were described as erosions or ulcers when examined endoscopically. We present a 47-year-old woman who presented with dysphagia. Endoscopy showed a lower esophageal fungating mass, mimicking a malignant mass. Although there was a high suspicion of esophageal carcinoma, biopsy results showed esophageal actinomyces infection.

## INTRODUCTION

Swallowing-associated problems affect 1 in 25 persons in the United States, and the prevalence increases with age. The estimated prevalence of dysphagia in patients older than 50 years is up to 22%.^1^ Well-known causes of obstructive esophageal dysphagia include esophageal stricture, esophageal carcinoma, eosinophilic esophagitis, and esophageal webs and rings. Upper endoscopy is an effective and essential tool for the evaluation of dysphagia, especially in men older than 40 years and/or when there is weight loss. Abnormal findings can be recognized by upper endoscopy in 70% of the patients present with dysphagia.^1^

Infectious esophagitis is the second most common cause of esophagitis after gastroesophageal reflux disease.^2^ Candida and herpes simplex virus and less commonly cytomegalovirus and human immunodeficiency virus (HIV) are the most frequently reported organisms that can cause infectious esophagitis. The usual endoscopic findings include esophageal erosions and ulcers, but the definitive diagnosis is only made after obtaining biopsies of the suspected lesions.

Esophageal actinomycosis (EA) is a rare esophageal infection that is generally found in immunocompromised patients only. EA is an extremely rare etiology of esophageal dysphagia. We present a case of EA mimicking esophageal malignancy in an immunocompromised host.

## CASE REPORT

A 47-year-old woman presented with progressive esophageal dysphagia. She has a past medical history significant for gastroesophageal reflux disease complicated by esophageal stricture status after dilation, type 2 diabetes mellitus, and chronic immunosuppressive therapy after a kidney-pancreas transplant about 7 years ago. Her dysphagia had been worsening lately and was associated with epigastric abdominal pain and weight loss. The patient denied heavy alcohol consumption or tobacco use. She had multiple esophageal dilations; the last was by balloon dilation about 3 months before her presentation. Physical examination was only significant for mild-epigastric tenderness.

**Figure 1.**
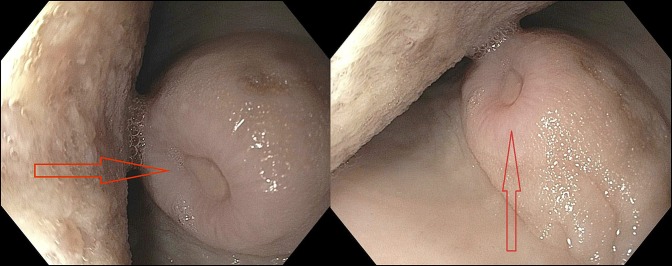
Endoscopy showing a fungating, ulcerated mid-esophageal mass (arrow).

The patient was anemic, with initial hemoglobin of 6.8 mg/dL. Chest computed tomography scan showed a patulous esophagus in the proximal and mid portions. The esophagus was decompressed in the distal portion with some degree of soft tissue thickening with no evidence of paraoesophageal lymphadenopathy. Endoscopy showed a fungating, ulcerated mid-esophageal mass, causing partial esophageal obstruction, concerning for esophageal carcinoma (Figure 1, arrow). EGD also showed few nonbleeding dispersed gastric erosions.

**Figure 2.**
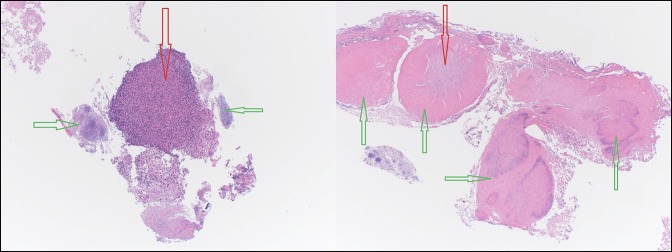
Biopsy of mid-esophageal mass show sulfur granules (green arrows) with the fibrinopurulent exudate (red arrows) in the center of them.

**Figure 3.**
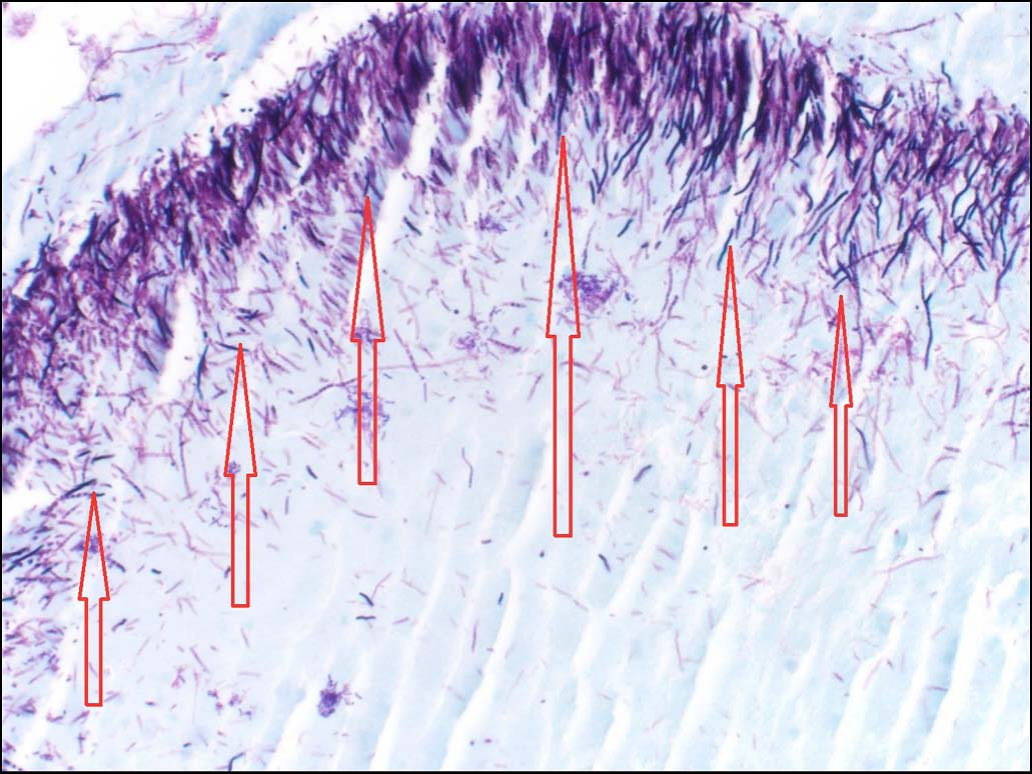
Gram staining of the sulfur granules showing the filamentous structures of the actinomyces (arrows).

**Figure 4.**
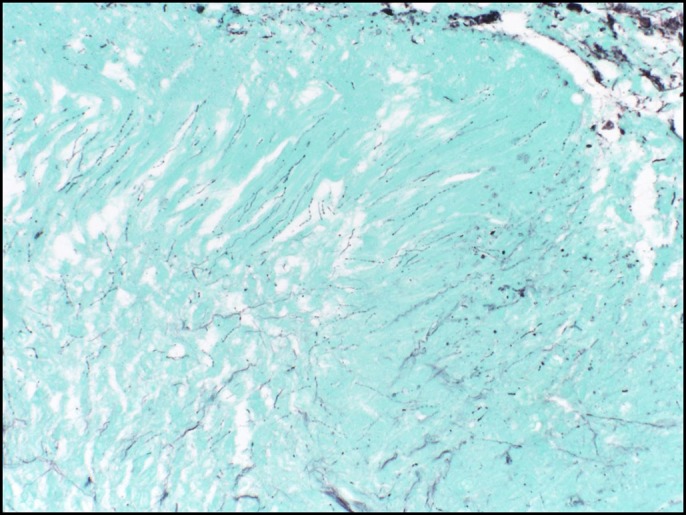
Grocott-Gomori's methenamine silver staining of the sulfur granules showing the filamentous structures of the actinomyces (dark areas).

The mass was biopsied during the procedure and the biopsy results showed fibrinopurulent exudate with bacterial overgrowth abundant with actinomyces, consistent with an esophageal actinomyces infection. There was no intact epithelium because of mucosal surface disruption. The sulfur granules and the fibrinopurulent exudate in the center of the sulfur granules is seen in the biopsy (Figure 2). When the sulfur granule was microscopically examined using the Gram stain (positively stained), the filamentous structures, which are a characteristic feature of the actinomyces, were shown (Figure 3). Similar features were seen using the Grocott-Gomori's methenamine silver stain, which shows the silver stain after being picked up by the filamentous actinomyces (dark areas) (Figure 4). There was no evidence of any malignant cells in the biopsy.

A following video capsule endoscopy and colonoscopy were unremarkable for similar lesions. The patient was started on intravenous penicillin for 8 weeks and was scheduled for a second-look endoscopy after this initial treatment course.

## DISCUSSION

Actinomyces are facultative anaerobic, Gram-positive bacilli. They commensally live within the oral cavity and gastrointestinal (GI) tract. They are slow-growing bacteria that can cause hard, pus-filled abscesses in the affected tissue.

EA is almost always preceded by mucosal disruption, which is necessary to facilitate the microorganism invasion, leading to various clinical senarios.^3^ The mucosal breakdown can result from infections and surgery or can be traumatic during endoscopic procedures. The appendix, cecum, and colon are the most common GI tract sites of actinomycosis infection, which can occur weeks to years after a mucosal disruption. For unexplained reasons, actinomycosis occurs more frequently in men than in women.^4^

The number of the reported cases has increased recently, but the incidence is still extremely rare with only about 24 cases of EA reported in the literature.^5^

The diagnosis of actinomyces infections can be challenging because isolation and identification of the bacteria occurs in only a minority of cases and radiologic findings are often nonspecific. Actinomyces culture frequently fails due to either previous antibiotic therapy, cross contamination with other oral and GI flora, or inadequate sampling. Microscopic examination is essential for diagnosis, which typically shows necrosis, yellowish “sulfur granules”, and filamentous Gram-positive fungal-like pathogens. The sulfur granules are centers of the organism colonies, surrounded by a rosette of clubbed filaments. A course of 4–6 weeks of antibiotics, typically with intravenous penicillin V, followed by a prolonged oral maintenance regimen is necessary for successful treatment. In some cases, surgical drainage may be required.

EA infection is an extremely rare cause of an esophageal mass lesion. Most previously described EA cases were noted to resemble esophagitis, esophageal ulcers, with the most commonly reported symptoms being odynophagia and dysphagia. Welling et al reported a case of a 27-year-old African-American man who presented with odynophagia.^6^ In that case, EGD showed an esophageal ulcerated lesion, which was found later to be EA. Arora et al and Chou et al reported cases of EA in association with esophageal candida infection.^7,8^ In both cases, the lesions were described as esophageal ulcerations. Nagaraju et al described a case of EA in a 28‐year‐old woman with end‐stage renal disease on regular hemodialysis.^9^ The endoscopic description of the EA lesion showed extensive esophageal necrotic areas.

A review of 15 cases of EA diagnosed on the basis of biopsies between 1970 and 2004 by Abdalla et al showed that the majority of the EA cases were associated with either malignancy or HIV (about half of the cases had HIV).^10^ EGD demonstrated esophageal ulceration in 10 of these 15 patients. Other reported EGD findings included granulation tissue, a traction diverticulum, plaques, strictures, and fistulas. Of note, the authors reported good clinical outcomes in response to the intravenous penicillin.

Clinicians should be aware that EA is a cause of dysphagia and odynophagia, particularly in immunocompromised patients and patients with HIV or malignancy. EA diagnosis remains difficult and requires a high index of suspicion and clinical knowledge regarding its unusual presentations and ability to mimic malignancy.

We present an extremely rare case of EA that likely resulted from the earlier traumatic esophageal dilation. This patient had no odynophagia due to the absence of the classic erosions or ulcers seen in other reported cases of EA. Her dysphagia resulting from the obstructive EA mass interestingly mimics esophageal malignancy, which makes it easy to misdiagnose.

## DISCLOSURES

Author contributions: A. Alshati wrote the manuscript and is article guarantor. L. Appleton, JT Maddux, and B. Dangerfield revised the manuscript. M. Almohammedawi edited the manuscript.

Financial disclosure: None.

Informed consent was obtained for this case report.
